# MicroRNAs as Biomarkers of Short-Term Complications After Cardiac Surgery

**DOI:** 10.3390/genes17030326

**Published:** 2026-03-17

**Authors:** Adam Kozik, Kamila Konstancja Kowalewska, Michał Piotrowski, Mariusz Kowalewski, Marian Burysz, Jakub Batko

**Affiliations:** 1 CAROL—Cardiothoracic Anatomy Research Operative Lab, Department of Cardiovascular Surgery and Transplantology, Institute of Cardiology, Jagiellonian University Medical College, 31-008 Krakow, Polandmarianburysz12@gmail.com (M.B.); jakub.batko@uj.edu.pl (J.B.); 2 Department of Cardiac Surgery and Transplantology, National Medical Institute of the Ministry of the Interior and Administration, 137 Wołoska Street, 02-507 Warsaw, Poland; 3 Thoracic Research Centre, Collegium Medicum, Nicolaus Copernicus University, Innovative Medical Forum, 85-067 Bydgoszcz, Poland; 4 Cardio-Thoracic Surgery Department, Heart and Vascular Centre, Maastricht University Medical Centre, 6229 HX Maastricht, The Netherlands; 5 Department of Cardiac Surgery, Regional Specialist Hospital, 86-300 Grudziądz, Poland; 6 Faculty of Medicine, Bydgoszcz University of Science and Technology, 85-796 Bydgoszcz, Poland

**Keywords:** microRNA, cardiothoracic surgery, cardiovascular interventions

## Abstract

Cardiac surgery carries substantial risk of early postoperative complications including postoperative atrial fibrillation (POAF, 30–50%), periprocedural myocardial infarction (PMI), acute kidney injury (AKI, 3.8–54.4%), bleeding (3–5%), stroke, and cognitive dysfunction. This narrative review synthesizes 30+ studies on circulating microRNAs (miRNAs) as perioperative biomarkers, identifying strongest evidence for cardiac-enriched miR-499 (AUC 0.93, sensitivity 85.7%, specificity 93.3%) and miR-133a (peaks 1–3 h post-declamping) in PMI diagnosis -outperforming troponins’ 6h kinetics. POAF prediction favors preoperative miR-483-5p (AUC 0.78), while AKI, bleeding (miR-223), and neurological injury show emerging but less validated candidates (miR-21, miR-210-3p). We critically analyze limitations across studies and outline clinical translation barriers (3–6 h assay times, heparin inhibition, lacking standardization) with solutions for point-of-care implementation.

## 1. Introduction

Cardiovascular diseases (CVD) are a heterogeneous group of conditions associated with dysfunction of the heart and vessels. They are one of the most common diseases worldwide, accounting for almost one third of global deaths, with stroke and heart attack being the most common causes [1]. CVD development is closely associated with age and several risk factors, including obesity, hypertension, smoking, diabetes, and high non-HDL cholesterol, which are more commonly observed nowadays due to lifestyle changes, which suggests that their incidence will steadily increase in the future [2,3].

Management of CVD involves lifestyle modifications, medication, and, in some cases, procedures—including both catheter and surgical options [4]. Nowadays, over a million cardiac surgeries are performed worldwide annually, with demand often exceeding the capacity of medical facilities [5]. The most common procedures include CABG and heart valve surgery, with aortic valve replacement being the most frequently performed [6]. Cardiac surgery is associated with various complications, including complications related to CPB, access site complications, and procedure-specific complications [6]. Among access-site complications after median sternotomy are wound-healing problems, including deep sternal infections with mortality rates reaching several percent, particularly in redo procedures or in diabetic patients [7,8,9]. Furthermore, the use of CPB triggers a systemic inflammatory response that can lead to multiorgan dysfunction [10]. CBP-related complications include pulmonary dysfunction, acute kidney injury (AKI) from systemic inflammation, vasoplegic syndrome, and neurological complications such as stroke, seizures or cognitive disturbances [10,11]. Procedure-specific complications after aortic valve replacement comprise paravalvular leak, prosthetic valve endocarditis, conduction disturbances requiring permanent pacemaker implantation, and vascular injury in transcatheter approaches [12]. In CABG, early graft failure, perioperative myocardial infarction (PMI), postoperative atrial fibrillation (POAF), and long-lasting impairment of pulmonary function and physical performance are among the most relevant adverse events [13]. Understanding these complications is essential for risk stratification and implementing preventive strategies to optimize patient management.

In everyday practice, the European System for Cardiac Operative Risk Evaluation (EuroSCORE II) or the Society of Thoracic Surgeons (STS) score is used for intraoperative mortality prediction in patients undergoing cardiac surgery [14]. Several studies evaluated its usefulness in the prediction of other complications [14,15,16]. With the development of surgery techniques and decreasing mortality during cardiac surgery, other life-impacting complications are being analyzed with growing interest [17]. Additionally, with the rapid growth of personalized medicine, patient-specific markers are under deep evaluation for the prediction and diagnosis of cardiac surgery complications. An example is microRNA (miRNA) [18]. Since their discovery in Caenorhabditis elegans in the early 1990s, miRNAs have emerged as central regulators of diverse biological processes including development, differentiation, cell proliferation, apoptosis, and cellular stress responses [19]. This review aims to evaluate available knowledge critically in regard to miRNA as a patient-specific marker of short-term complications related to cardiac surgery.

## 2. Complications After Cardiac Surgery

As mentioned earlier, application of CBP sets cardiac surgery apart from other surgical disciplines and is associated with a distinct spectrum of potential postoperative complications. Among the most common, we can distinguish PMI, stroke and cognitive dysfunction, AKI, POAF, bleeding and sternal wound infection [20].

### 2.1. Perioperative Myocardial Infarction

Myocardial infarction (MI) continues to be a major cause of death in developed countries. Worldwide, an estimated three million people experience MIs each year. MI occurs when the heart muscle does not receive enough oxygen for a prolonged period, leading to permanent damage. This injury can weaken both the systolic and diastolic functions of the heart and increase the likelihood of developing arrhythmias. A wide range of acute and chronic complications may follow, underscoring the need for rapid intervention. Restoring blood flow as quickly as possible remains critical for improving survival and long-term outcomes [21,22,23].

### 2.2. Stroke and Cognitive Dysfunction

Stroke may occur during or after cardiac surgery and is generally divided into intraoperative stroke, mainly due to aortic manipulation or atheroembolism, and delayed postoperative stroke, typically related to POAF or underlying cerebrovascular disease [24]. Stroke prevention in this setting remains difficult because current prediction tools, based on clinical risk factors, imaging, and perioperative monitoring, have limited sensitivity and often miss high-risk individuals. The most up-to-date preventive measures include minimizing aortic manipulation, optimal CPB management, strict blood pressure control, sufficient anticoagulation, and prompt treatment of POAF with β-blockers or amiodarone [20]. Yet, postoperative stroke still occurs, highlighting the need for better risk-stratification strategies.

### 2.3. Acute Kidney Injury

AKI is a reversible reduction in kidney function that leads to the accumulation of metabolic waste products [25]. It is a frequent and serious complication after cardiac surgery, associated with high in-hospital mortality (3.8–54.4%) and the need for renal replacement therapy in 2–5% of cases [26,27].

According to KDIGO guidelines, AKI is diagnosed based on changes in serum creatinine or urine output; however, both markers are delayed and nonspecific, often rising only after significant renal injury has already occurred, creating an urgent need for earlier and more specific biomarkers [28]. Causes include ischemia–reperfusion injury, sepsis, nephrotoxins, and immune-mediated damage, with cardiac surgery-associated AKI driven largely by renal hypoperfusion, CPB-related hemodynamic alterations, hemolysis, and systemic inflammation [29].

Despite preventive strategies based on KDIGO bundles (optimization of hemodynamics and fluid balance, limiting nephrotoxins, controlling hyperglycemia, and adjusting drug doses), AKI still occurs in 3.8–54.4% of patients, underlining the limitations of current approaches and the diagnostic gap of creatinine and urine output [28].

### 2.4. Postoperative Atrial Fibrillation

Atrial fibrillation (AF) is the most frequently diagnosed arrhythmia in the world among adults [30]. It can lead to serious complications such as stroke and heart failure and significantly impairs the quality of life [31,32]. It is also one of the most common arrhythmias after cardiac surgery, affecting approximately 30–50% of patients, having a direct impact on increased mortality and more frequent occurrence of complications such as stroke, MI and heart failure [33,34].

POAF most often appears in the first few days after surgery [35]. It is worth noting that, due to the high risk of bleeding, traditional approaches such as oral anticoagulants (OACs) therapy have limited applicability in the immediate postoperative period [36,37,38]. Anticoagulant therapy after high-bleeding-risk procedures such as CABG should be initiated 48 h after surgery in patients with a clear indication for its use, provided that hemostasis is secure. In patients with AF, bridging therapy (the use of a short-acting anticoagulant during temporary interruption of OACs) is generally discouraged [39]. Overall, evidence indicates that while OACs reduce thromboembolic risk in the setting of cardiac surgery, they simultaneously increase the risk of bleeding [37].

The pathophysiology of POAF is complex, and its occurrence is a combination of multiple factors, such as preexisting degenerative changes in the atrial myocardium or perioperative conditions that influence the development of conduction disturbances [40].

Although some patients may develop POAF without any apparent risk factors, at least one risk factor is often present. The most common include advanced age, diseases such as obesity, diabetes, right circumflex artery stenosis, a history of AF, and prior cardiac surgery [41,42,43,44,45].

### 2.5. Bleeding

Perioperative hemorrhage and postoperative thromboembolism are two seemingly paradoxical complications associated with surgical interventions [20]. Strikingly, many surgical patients simultaneously face both bleeding and clotting risks within the first 24–72 h after surgery—a phenomenon termed the hemostatic paradox. This problem illustrates the need to maintain a complex balance between procoagulant factors (like tissue factor, thrombin, platelets), anticoagulant mechanisms (proteins C and S, antithrombin, tissue factor pathway inhibitor) and fibrinolytic systems (plasmin, tissue plasminogen activator, plasminogen activator inhibitor-1) [46].

Peri- and postoperative bleeding represents one of the most serious and challenging complications of surgical procedures, particularly in cardiac surgery, and is associated with increased morbidity, mortality, prolonged hospitalization, and substantial healthcare costs [47]. Severe bleeding, requiring transfusion of more than 10 units of packed red blood cells, is reported in approximately 3–5% of patients after CPB [48]. Most guidelines do not recommend routine assessment of the risk of bleeding through laboratory tests in all patients, but only in selected populations such as those with history of bleeding or bleeding disorder in the family [49,50,51,52]. Current methods for predicting perioperative bleeding risk rely primarily on clinical scoring systems such as SHOULD-NOT-BLEED, WILL-BLEED, Papworth or TRUST risk scores [49,50,51,52]. However, these scoring systems demonstrate limited discriminatory power and do not account for the complex molecular and cellular mechanisms underlying haemostatic dysfunction.

### 2.6. Sternal Wound Infection

Deep sternal wound infection (SWI) is a major postoperative complication that happens after sternotomy. It is a surgical procedure where the surgeon cuts through the sternum to provide access to the heart or lungs. It is often used in procedures such as bypass, valve repair, or transplants [53]. When pathogens from the incision penetrate deeper tissues, it may initiate a large-scale immune response with cytokine release, fever, leukocytosis or leukopenia, tachycardia and tachypnea, fulfilling the criteria for systematic inflammatory response syndrome (SIRS). It can cause multiorgan failure, sepsis and eventually death. Furthermore, severe cardiac procedures involving CPB often start systematic inflammatory reactions—this impairs immune balance, microcirculation and wound healing, therefore rising vulnerability to SWI [54]. Thus, SIRS and SWI are interlinked: SWI may cause SIRS, while surgery-induced SIRS can predispose to SWI, creating a malignant cycle that raises postoperative morbidity and mortality [9].

## 3. miRNA Detection

miRNAs can be detected in virtually all biological fluids, including plasma, serum, cerebrospinal fluid, saliva, and urine, making them a potentially easy-to-obtain biomarker candidate [55,56]. The presence of miRNAs in these extracellular compartments was initially unexpected due to their easy degradation by endonucleases. However, miRNAs present in body fluids appear to resist fast degradation. Endogenous miRNAs present in plasma are not degraded as readily as their synthetic counterparts due to the existence of appropriate transport systems [57]. Studies have revealed the existence of two distinct populations of circulating miRNAs [58]. One is associated with vesicles—exosomes, microvesicles, or apoptotic bodies—where the membrane bilayer of these vesicles provides protection for the enclosed miRNAs from extracellular RNases [58,59,60]. The other is associated with specific proteins, such as Ago2, which also perform protective functions [58].

An important aspect to consider when using miRNAs as biomarkers is their stability. Unfortunately, they exhibit widely variable stability kinetics dependent on intrinsic sequence properties, secondary structure, and association partners (like Ago2) [58,60]. Under physiological conditions, they exhibit half-lives ranging from several to a dozen or so hours. However, Katayama et al., using miR-15b, miR-16, miR-21, miR-24, and miR-223 as examples, demonstrated their high stability after 24 h of storage at room temperature and on ice [61]. Research involving a small number of miRNAs indicates that plasma miRNAs remain largely stable during both short- and long-term storage at −80 °C. For instance, analyses of eight miRNAs from five healthy individuals demonstrated strong stability and an extended frozen half-life (ranking from 6 to 12 months and up to 14 years) in plasma [62]. miRNAs with biomarker potential may serve both diagnostic and predictive roles, with the most relevant examples summarized in Table 1 and Table 2.

## 4. miRNA as Diagnostic Markers

### 4.1. Myocardial Infarction and Perioperative Myocardial Injury (PMI)

Despite multiple studies related to miRNAs in MI, limited data about their role in diagnosis of PMI after cardiac surgery is available. Yao and colleagues reported that circulating cardiac-enriched miRNAs rise rapidly after on-pump CABG and may outperform traditional biomarkers in the early detection of perioperative myocardial infarction. In their cohort, miR-499, miR-133a, and miR-133b peaked within 1–3 h after aortic declamping—well before the 6 h peak observed for cTnI—and their peak concentrations correlated strongly with the extent of myocardial injury. The authors also found that patients undergoing off-pump CABG exhibited markedly lower postoperative miRNA levels, supporting their specificity for ischemic myocardial damage. The best independent factor for PMI in that study was miR-499 with sensitivity of 85.7% and specificity of 93.3%. The authors suggested that miR-499 represents a promising early biomarker for PMI in cardiac surgery [71,72].

### 4.2. Stroke and Neurological Dysfunction

In the context of neurological complications, Szwed et al. investigated whether circulating miRNAs could predict neurological dysfunction. In 30 elective off-pump CABG patients, plasma levels of miR-21-5p (AUC 0.778), miR-1-3p, and GFAP were measured perioperatively. Early postoperative rises in miR-21-5p and GFAP were strongly associated with early postoperative cognitive dysfunction, validating the potential of miRNAs as diagnostic biomarkers for perioperative neurological injury [76]. Furthermore, Sultan et al. suggested that blood-based miRNAs may be useful as non-invasive biomarkers for early detection of intracerebral hemorrhage. They identified 34 miRNAs associated with poor prognosis, notably miR-126 and miR-23a-3p, whose expression levels correlated with the volume of peri-hematomal edema, indicating they may reflect edema severity [77].

### 4.3. Acute Kidney Injury (AKI)

For AKI, several miRNAs have been explored as molecular markers. Wang et al. reported elevated urinary, but not serum, miR-10a 24 h after renal ischemia–reperfusion in mice. Quantitative PCR showed that miR-10a surges within 1 h after reperfusion, peaks at 6 h, and stays elevated compared to controls, appearing as a favorable AKI biomarker due to its rapid response and renal specificity. Other miRNAs, such as miR-101-3p, have proven practical in diagnosing AKI in ICU patients. Additionally, miR-210-3p (AUC 0.935) and miR-146a-5p (AUC 1.000) have been linked to post-AKI mortality in Intensive Care Unit patients, correlating with disease severity [78].

### 4.4. Postoperative Atrial Fibrillation (POAF)

To date, several models have been developed that consider several clinical parameters, which, however, are not routinely used in clinical practice [35,79]. Many methods have been developed to prevent this complication [36]. There are also reports indicating that certain treatment strategies, such as off-pump CABG, posterior pericardiotomy, and preservation of the anterior fat pad, constitute potential negative risk factors [80,81,82].

As early as 2005, Barth et al. observed significant changes in gene expression in atrial cells in patients with persistent AF, which could indicate a substantial contribution of regulatory mechanisms to the pathogenesis of the disease [83]. The role of miRNA in AF was observed in regulation of several factors, including fibrosis, inflammation, and oxidative stress [84]. However, the role of miRNAs in POAF remains relatively poorly understood, and many available studies focus on their involvement in the mechanisms contributing to the development of the disease rather than on their predictive potential.

Feldman et al. observed decreased levels of miR-23a and miR-26a in patients who developed POAF after CABG. However, the observed changes were only visible after the procedure, which indicates their potential as a diagnostic biomarker [70]. Also, there are reports that its level is higher in right atrial cells in patients developing POAF after cardiosurgery [64]. The authors observed increased expression of miR-1 and decreased expression of miR-133A in the examined tissue. Importantly, miR-1 acts as a proapoptotic factor in cardiomyocytes, while miR-133A acts as an antiapoptotic factor [85]. At the same time, the authors observed increased activity of apoptosis markers in the RAA, such as TUNEL, caspase-3, and mRNA for Bcl2 and Bax [64].

Unfortunately, to date, the evidence regarding the role of microRNAs in POAF comes primarily from studies conducted in patients undergoing CABG surgery, while research in other disease entities remains limited.

While numerous miRNAs have been identified as potential biomarkers across various complications, the strength of evidence and clinical readiness vary significantly between them. A cross-complication comparison reveals that the most robust evidence currently supports the use of cardiac-enriched miRNAs, specifically miR-499 and miR-133a, for the diagnosis of PMI. Meta-analyses consistently demonstrate that these biomarkers offer superior diagnostic accuracy compared to novel markers for other complications, with miR-499 showing pooled sensitivity and specificity exceeding 85% and 90%, respectively. Furthermore, their kinetic profile provides a distinct advantage over traditional high-sensitivity troponins, as they peak significantly earlier (1–3 h after aortic declamping), potentially narrowing the diagnostic window in the critical early postoperative period [86].

In contrast, the evidence for biomarkers predicting postoperative atrial fibrillation (POAF) and acute kidney injury (AKI) is more moderate. While miR-483-5p shows promise as a preoperative predictor for POAF, its utility is primarily risk stratification rather than acute diagnosis, offering a diagnostic accuracy of approximately 78%. The least robust biomarkers appear to be those for bleeding and neurological complications; candidates like miR-223 for bleeding rely on smaller, isolated cohorts and lack the multi-center validation required to compete with standard coagulation panels [87].

From a practical standpoint, the clinical utility of these markers is heavily stratified by the urgency of the complication. For acute events like PMI or massive hemorrhage, where clinical decisions must be made within minutes, the current turnaround time of standard PCR assays is a limiting factor compared to point-of-care coagulation tests or rapid troponin assays. Conversely, for POAF and AKI prediction, where risk stratification occurs preoperatively or in the sub-acute phase, the longer processing time of miRNA assays is less prohibitive, making these candidates more immediately viable for clinical translation than acute event markers [88].

## 5. MiRNAs as Predictor of Short Term Complications

Risk stratification is an essential component of modern cardiovascular care, allowing for early identification of patients at increased risk of adverse postoperative events. Accurate risk assessment supports personalized therapeutic decisions and targeted monitoring strategies. Incorporating robust stratification models into clinical practice contributes to improved outcomes and more efficient resource allocation.

### 5.1. Myocardial Infarction

miR133a, which proved to be good diagnostic tool in PMI detection, was also investigated in percutaneous coronary intervention (PCI). A study conducted by Zhou and colleagues found that levels of miR-133a before and after intervention were predictors of PMI with a sensitivity of 93.8% and a specificity of 71.9% [51]. Presumably, miR133a and diagnostic molecules mentioned earlier (miR-499, miR-133b) might be investigated in further prospective studies to clarify their capacity for predicting and diagnosing PMI after cardiac surgery.

### 5.2. Acute Kidney Injury

In the context of identifying non-invasive predictive biomarkers for AKI, increasing attention has been focused on compartment-specific alterations in miRNA expression, as demonstrated by the findings reported by Saikumar et al. They reported reduced miR-21 levels in peripheral blood and increased urinary levels after an AKI incident in a rat model, with an AUC-ROC value in patients of 0.71 [89].

### 5.3. Postoperative Atrial Fibrillation

In the context of currently used biomarkers in POAF, unfortunately, none offer sufficient predictive value to reliably assess the risk of postoperative AF with high sensitivity and specificity.

One miRNA that constitutes a potential biomarker is miR-483-5p. Harling et al. demonstrated that its high levels in both serum and atrial cardiomyocytes increase the risk of developing POAF in patients undergoing CABG, with sensitivity and specificity of 78% and 77%, respectively [63]. miR-483-5p is co-transcribed with the host gene *IGF2* [90]. It has been postulated that overexpression of *IGF2* may be responsible for regulating pro-inflammatory pathways connected to NF-kB and IL-6, which in turn may promote the development of AF following surgery [91,92].

Sathipati et al. analyzed a panel of 84 plasma miRNAs, identifying changes in the expression of 13 of them among patients who underwent CABG. hsa-miR-200a-3p, hsa-let-7a-5p, and hsa-miR-423-5p appeared to have the most predictive value. Their involvement in signaling pathways dysregulated in these diseases, such as MAPK, PI3K-Akt, FoxO, and TGF-beta, was confirmed [93].

The role of miR23 in the pathophysiology of cardiac conduction disorders has not been well understood yet. Some studies indicate its role in processes such as cardiac valve formation, atrial fibrosis, or the regulation of cardiomyocyte hypertrophy [94,95,96]. The authors also examined differences in miR-1 levels, finding no statistically significant differences [97].

Another potential biomarker appears to be miR-199a and SIRT1 protein, the expression of which was assessed in patients undergoing CABG. Unfortunately, the study material consisted of atrial cardiomyocytes, which significantly limits their predictive potential. A significant reduction in miR-199a and an increase in SIRT1 protein levels were found in the group of patients who developed POAF [65]. Importantly, SIRT1 regulates a key hypoxia response pathway associated with HIF-1 [98]. Oxidative stress, in turn, increases the risk of developing AF [99].

An interesting approach seems to be searching for markers by examining the content of exosomes. The miRNA molecules contained in them are characterized by higher stability, which may make them a better target as a potential biomarker [100]. miR-122-5p was identified as a potential exosomal biomarker that can be used to assess the risk of developing POAF in patients undergoing CABG [66]. Additional analysis of its target genes showed that this miRNA may influence atrial structure through many signaling pathways in POAF pathogenesis [66]. In turn, other studies have indicated that this miRNA is elevated in the AF mice model and likely promotes cardiomyocyte apoptosis [101].

### 5.4. Bleeding

In the context of bleeding risk prediction, only a limited number of studies have investigated the potential utility of miRNA as biomarkers. The key miRNA that can be used in the risk assessment of bleeding is miR-223. Wang et al. showed that higher circulating miR-223 levels independently predicted severe perioperative bleeding in patients undergoing CABG [67]. Additionally, in the same study, it was noted that among patients treated with ticagrelor, the level of miR-223 was higher than in the clopidogrel-treated group, which may indicate that more potent P2Y12 inhibition upregulates circulating miR-223 [67].

### 5.5. Systemic Inflammation and Wound Infection

In the context of systemic inflammatory response and potential wound infection complications, Wagner et al. discovered that plasma levels of myocardial miR-133a rapidly increased after cardiac procedures. Patients who underwent intraoperative cytokine hemadsorption had a greater rise in miR-133a, suggesting that modulation of the inflammatory response can unexpectedly affect myocardial injury markers [69].

## 6. Comparative Analysis of Technical Data

A systematic evaluation of the reviewed literature reveals substantial methodological heterogeneity and design limitations that currently hinder the translation of miRNA biomarkers into routine clinical practice (Table 3). Most identified studies are single-center pilot investigations with small sample sizes (*n* < 100), often lacking external validation cohorts. For instance, studies by Yao et al. and Szwed et al. relied on groups as small as 30 patients, which limited the generalizability of their findings to broader surgical populations.

Moreover, the diversity of biological matrices employed across studies precludes direct comparison of results. While circulating miRNAs in plasma or serum are preferred for non-invasive testing, some investigations, such as those by Yamac et al. and Harling et al., were based on tissue analysis [65,91]. Although tissue-based findings provide mechanistic insights, they are of limited utility for pre-operative risk stratification in a clinical setting. Additionally, reliance on animal models in AKI research conducted by Saikumar et al. or Wang et al., introduces a translational gap, as rodent models of renal ischemia–reperfusion do not fully replicate the complex physiology of a human [67,89].

Another critical source of bias is the variability in study populations and surgical techniques. Several key studies, including those by Wang et al. (bleeding) and Szwed et al. (stroke), focused exclusively on OPCAB patients. Since CPB is a major driver of systemic inflammation, hemolysis, and coagulopathy, biomarkers validated in OPCAB cohorts may not be applicable to on-pump procedures [67,76]. Similarly, data extrapolated from non-surgical cohorts, such as PCI patients (Zhou et al.) or spontaneous intracerebral hemorrhage (Sultan et al.), may not accurately reflect the specific pathophysiology of perioperative complications [63,77].

Finally, the timing of sampling varies significantly, distinguishing diagnostic markers from truly predictive ones. Studies such as Feldman et al. relied on post-operative sampling, which, while useful for diagnosis, fails to aid in preoperative risk stratification and preventive decision-making [70].

Consequently, large-scale, multi-center studies utilizing standardized protocols and uniform time-points are urgently needed to validate these preliminary findings.

## 7. Challenges

Despite significant progress made in recent years in understanding miRNA biology and their potential role as biomarkers, significant challenges remain before miRNA-based technologies can be widely used in the clinic [102]. One of the key factors appears to be the need to standardize procedures for sample collection and storage, as well as for determining biomarker levels [102]. Studies have shown that the recorded miRNA levels are influenced by, among other things, the choice of anticoagulant—EDTA, citrate, or heparin [97,103]. Moreover, each potential biomarker requires careful evaluation, as factors unrelated to the disease, such as physical activity or circadian rhythm, can influence its level. Hemolysis is an additional factor, as some miRNAs can be released from erythrocytes [104]. A recent technical note by the European Atherosclerosis Society proposed minimum pre-analytical information requirements for the publication of circulating miRNA studies, including standardized reporting of collection timing, sample type, centrifugation protocols, hemolysis assessment, and documentation of potential confounding variables [105]. It is also worth emphasizing the lack of standardized protocols regarding isolation methods. No universally accepted “gold standard” extraction protocol exists for circulating miRNAs, and the optimal method may vary depending on the downstream application (qRT-PCR, microarray, RNA-seq) and the specific miRNAs of interest [106]. Furthermore, one of the greatest challenges remains the limited number of available studies—most of them focus on CABG, and the results remain inconsistent. Similarly problematic is the definition of “upregulated” and “downregulated,” which is often arbitrarily determined, usually relative to the median in a given study, rather than as an objectively adopted cutoff value. Beyond the pre-analytical variables regarding sample collection, widespread clinical adoption of miRNA biomarkers faces substantial hurdles regarding cost, assay turnaround time, and biological standardization. The economic burden of miRNA profiling remains a primary impediment; standard quantitative real-time PCR (qRT-PCR) and Next-Generation Sequencing (NGS) platforms are significantly more resource-intensive than routine biochemical assays such as serum creatinine or INR used in current practice. For a novel biomarker to replace or potentialize a standard test, it must demonstrate not just clinical non-inferiority but also cost-effectiveness, a threshold that has not yet been met for routine perioperative screening [107].

Furthermore, a critical “diagnostic gap” exists between assay speed and clinical urgency. Perioperative complications often require immediate decision-making, yet standard miRNA extraction and amplification protocols typically require 3 to 6 h, rendering them impractical for intraoperative or acute postoperative management. While emerging Point-of-Care technologies utilizing microfluidics or paper-based biosensors promise results in under an hour, these platforms are not yet commercially available or validated for the complex matrix of cardiac surgical patients [88].

Finally, the lack of assay standardization remains a significant barrier to real-world implementation. Unlike established metabolites, there is no consensus on “gold standard” normalization controls for circulating miRNAs, and inter-platform variability between microarray and PCR results complicates data synthesis. This is exacerbated in cardiac surgery by the routine use of high-dose heparin, a potent inhibitor of PCR enzymes, which can lead to false-negative results if not meticulously removed during the RNA extraction process. Additionally, biological variability due to patient-specific factors, such as circadian rhythms and physical activity levels, can alter baseline miRNA expression, further complicating the establishment of universal reference intervals essential for clinical interpretation [88].

## 8. Conclusions

While numerous miRNAs have been identified as potential biomarkers across various complications, the strength of evidence and clinical readiness vary significantly between them. A cross-complication comparison reveals that the most robust evidence currently supports the use of cardiac-enriched miRNAs, specifically miR-499 and miR-133a, for the diagnosis of PMI. Meta-analyses consistently demonstrate that these biomarkers offer superior diagnostic accuracy compared to novel markers for other complications, with miR-499 showing pooled sensitivity and specificity exceeding 85% and 90%, respectively. Furthermore, their kinetic profile provides a distinct advantage over traditional high-sensitivity troponins, as they peak significantly earlier (1–3 h after aortic declamping), potentially narrowing the diagnostic window in the critical early postoperative period. In contrast, the evidence for biomarkers predicting POAF and AKI is more moderate. While miR-483-5p shows promise as a preoperative predictor for POAF, its utility is primarily risk stratification rather than acute diagnosis, offering a diagnostic accuracy of approximately 78%. The least robust biomarkers appear to be those for bleeding and neurological complications; candidates like miR-223 for bleeding rely on smaller, isolated cohorts and lack the multi-center validation required to compete with standard coagulation panels [86].

## Figures and Tables

**Table 1:** miRNA as predictive markers of short-term complications after cardiac surgery.

Disease	Potential Biomarker	Expression Pattern	Sample Type and Size	Intervention	Time of Measurement	AUC, Sensitivity, Specificity	References
Predictive function							
POAF	miR-483-5p	↑	Serum, atrial cardiomyocytes; 34	CABG	Before intervention	0.78; 77%; 78%	[63]
	miR-1	↑	Serum, atrial cardiomyocytes; 42	CABG	Before intervention	N/A	[64]
	miR-199a	↓	Atrial cardiomyocytes, 63	CABG	During intervention	N/A	[65]
	exosomal miR-122-5p	↑	Serum, 12	CABG	Before intervention	0.8; 100%; 60%	[66]
Bleeding	miR-223	↑	Serum, 59	CABG	Before intervention	N/A	[67]
AKI	miR-21	↑	Serum, 115	CABG, valve replacement	Before intervention	0.7	[68]
Inflammatory response	miR-133a	↑	Serum, 32	David or Ross procedure	Before and after intervention	N/A	[69]

↑ and ↓ indicate upregulated and downregulated expression patterns, respectively.

**Table 2:** miRNA as diagnostic markers of short-term complications after cardiac surgery.

Diagnostic Function							
POAF	miR-23a and miR-26a	↓	Serum, 48	CABG	After intervention	0.63 and 0.67	[70]
	miR-133a	↓	Atrial cardiomyocytes, 42	CABG	After intervention	N/A	[64]
MI	miR-499, miR-133b	↑	Serum, 30	CABG	After intervention	0.93; 85%; 92%	[71,72]
	miR-133a	↑	Serum	CABG, PCI	Before and after intervention	N/A	[63,72]
AKI	miR-101-3p	↑	Serum	N.A.	After onset of symptoms	N/A	[73]
	miR-210-3p	↑	Serum, 66	N.A.	After onset of symptoms	0,7	[74]
	miR-146a-5p	↑	Serum, 35	N.A.	After onset of symptoms	1	[75]
Cognitive dysfunction	miR-21-5p	↑	Serum, 30	Off-pump CABG	During and after intervention	N/A	[76]
Intracerebral hemorrhage	miR-126, miR-23a-3p	↑	Serum, 35	N.A.	After onset of symptoms	0,98	[77]

↑ and ↓ indicate upregulated and downregulated expression patterns, respectively.

**Table 3:** Summary of miRNA studies evaluating predictive biomarkers of major complications in cardiac surgery.

References	Complication	Study Type/Population	Sample Size (*n*)	Methodology& Matrix	Critical Limitations (Risk of Bias)
[72]	PMI	Clinical (CABG)	*n* = 30	qRT-PCR (Plasma)	Single-center; small sample; limited validation window.
[63]	PMI	Clinical (PCI)	*n* = 80	qRT-PCR (Serum)	Not cardiac surgery (PCI cohort); extrapolation to CABG is indirect.
[91]	POAF	Clinical (CABG)	*n* = 34 (13 POAF)	qRT-PCR (Serum & Tissue)	Small sample; exploratory design; mixed tissue/serum findings.
[70]	POAF	Clinical (CABG)	*n* = 60	qRT-PCR (Serum)	Post-operative sampling only (diagnostic, not predictive); single-center.
[65]	POAF	Clinical	*n* = 44	qRT-PCR (Tissue)	Tissue only (atrial cardiomyocytes); cannot be used as a non-invasive blood biomarkers.
[93]	POAF	Clinical (CABG)	*n* = 15	qRT-PCR Array (Plasma)	High-throughput screen but very small validation set; high risk of false positives.
[68]	AKI	Clinical (CABG)	*n* = 115	qRT-PCR (Serum)	Variable definition of AKI; modest AUC (0.70); single-center.
[89]	AKI	Animal Model (rats)	Unknown	qRT-PCR (Blood)	Animal model; translational gap to human CPB physiology is significant.
[78]	AKI	Animal Model (mice)	Unknown	qRT-PCR (Plasma)	Mouse models of renal ischemia; kinetics may not reflect human CPB injury.
[67]	Bleeding	Clinical (OPCAB)	*n* = 90	qRT-PCR (Plasma)	Restricted to Off-Pump cases (no CPB effect); results may not apply to on-pump surgery.
[76]	Stroke	Clinical (OPCAB)	*n* = 30	qRT-PCR (Plasma)	Pilot study size; OPCAB only (no CPB embolism risk); short follow-up.
[77]	Stroke (ICH)	Clinical (ICH)	*n* = 87	qRT-PCR (Blood)	Spontaneous ICH patients, not perioperative cardiac surgery strokes; different etiology.
[69]	Inflammation	Clinical	*n* = 28	qRT-PCR (Plasma)	Extremely small sample; confounded by cytokine hemoadsorption intervention.

## Data Availability

No new data were created or analyzed in this study.

## Abbreviations

The following abbreviations are used in this manuscript:

AF	atrial fibrillation
AKI	acute kidney injury
CABG	coronary artery bypass grafting
CPB	cardiopulmonary bypass
CVD	cardiovascular diseases
microRNA	miRNA
MI	myocardial infarction
PARN	poly(A)-specific ribonuclease
PCI	percutaneous coronary intervention
PMI	perioperative myocardial infarction
POAF	postoperative atrial fibrillation
Pol II	RNA polymerase II
RISC	RNA-induced silencing complex
STS	Society of Thoracic Surgeons
SWI	sternal wound infection
